# Acute kidney injury and iron metabolism: A narrative review focusing on pathophysiology and therapy

**DOI:** 10.1002/smo.20240026

**Published:** 2025-01-07

**Authors:** Xinyu Tan, Man Li, Jing Chen, Gang Liu, Zhixiang Lu

**Affiliations:** ^1^ School of Life Sciences Xiamen University Xiamen China; ^2^ Department of Child Health Women and Children's Hospital School of Medicine Xiamen University Xiamen Fujian China; ^3^ State Key Laboratory of Molecular Vaccinology and Molecular Diagnostics Center for Molecular Imaging and Translational Medicine School of Public Health Xiamen University Xiamen China; ^4^ School of Pharmaceutical Sciences Xiamen University Xiamen China

**Keywords:** acute kidney injury, biomarker, catalytic iron, iron homeostasis, translational research

## Abstract

Acute kidney injury (AKI) represents a substantial challenge to public health and is characterized by elevated occurrence and fatality rates. In the last 3 decades, the disruption of iron homeostasis and the cytotoxic effects mediated by iron have been extensively acknowledged as contributors to, as well as outcomes of, renal damage. Therefore, iron metabolism has become the focus of novel therapeutic interventions for AKI, with targeted iron metabolism strategies showing great potential. In this review, we have explored the dysregulation of iron metabolism in AKI and the AKI caused by iron metabolism disorders. We have summarized the complex mechanisms of iron metabolism in the kidney and emphasized the potential role of iron metabolism‐related metabolic pathways in the treatment and prevention of AKI. Finally, we have reviewed various strategies targeting iron metabolism for the treatment of AKI, hoping to provide more effective treatment options for AKI patients in the future.

## Graphical abstract

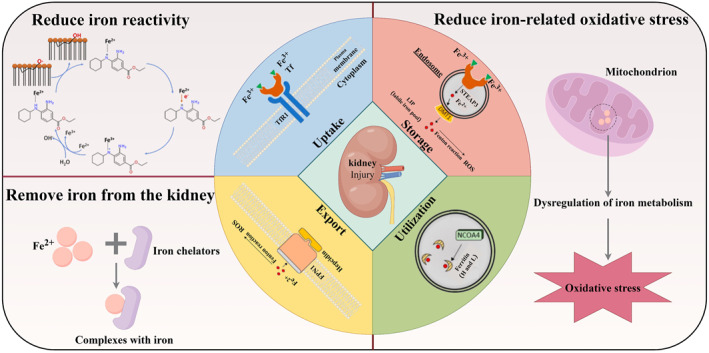

## 1 INTRODUCTION

Acute kidney injury (AKI) is a medical syndrome in which the functionality of the kidneys decreases rapidly and over a short period of time, mainly characterized by a sharp decrease in glomerular filtration rate and rapid accumulation of nitrogenous wastes in the blood.^[1]^ Kidney Disease: Improving Global Outcomes defines AKI as kidney disease with an absolute increase in serum creatinine (sCr) of ≥3 mg/L or an increase over baseline of 50% within 48 h or 7 days.^[2]^ The pathological manifestations of AKI are diverse, with typical changes including tubular cell necrosis and detachment, significant tubular dilation, and infiltration of inflammatory cells.[^3^, ^4^]

AKI is a common complication of acute illnesses, which can lead to multi‐organ failure and even death in severe cases. Recognized worldwide as a difficult‐to‐treat disease, AKI affects 2%–7% of hospital admissions and more than 35% of patients admitted to intensive care.[^5^, ^6^] Currently, the treatment options for AKI are limited, mainly focusing on fluid resuscitation and renal replacement therapy (RRT), with RRT being the primary supportive treatment for patients with severe AKI.^[7]^ Five to six percent of critically ill patients require this treatment method, yet the hospital mortality rate remains high at 50%–80%.[^8^, ^9^] After years of research, it has been found that there is still a global lack of effective and specific drugs for the treatment of AKI.^[10]^ Consequently, there is a pressing demand to investigate the pathological environment of AKI, and then implement effective pharmacological interventions, which would hold substantial clinical significance and research relevance.

The pathogenesis of AKI is quite complex and is currently mainly divided into three categories: prerenal, intrinsic renal, and postrenal.^[11]^ Prerenal AKI is primarily caused by insufficient kidney perfusion due to factors such as gastrointestinal bleeding, hepatorenal syndrome, renal artery stenosis or thrombosis, and heart failure, leading to a decrease in glomerular filtration rate.^[12]^ Intrinsic renal AKI mainly involves damage to the glomeruli, tubules, and interstitium, and can be seen in conditions like primary nephrotic syndrome, acute and chronic glomerulonephritis, sepsis, rhabdomyolysis, and the use of nephrotoxic drugs such as nonsteroidal anti‐inflammatory drugs, antimicrobials, angiotensin‐converting enzyme inhibitors, angiotensin II receptor blockers, and iodinated radiocontrast agents, as well as major surgical procedures like heart surgery and kidney transplantation.^[13]^ Urinary tract obstruction is the primary cause of postrenal AKI.^[14]^ Recent investigations indicate that iron is considerably involved in the development of AKI.^[15]^ At physiological concentrations, iron is an essential nutrient for the human body, performing a variety of important biological functions. However, both excess and deficiency of iron within the body play crucial roles in the development of AKI. Moreover, numerous studies have demonstrated a link between ferroptosis and AKI. Ferroptosis, identified as a novel and iron‐dependent form of regulated cell death, is increasingly recognized for its role in AKI. Evidence suggests that by targeting and inhibiting ferroptosis, the severity of AKI can be effectively alleviated.[^16^, ^17^, ^18^, ^19^] Given the significant gaps in our current understanding and the potential implications for the development of novel therapeutic approaches, the exploration of the specific regulatory mechanisms of ferroptosis in AKI is poised to become a prominent area of focus in future medical and scientific research.

**Scheme 1:**
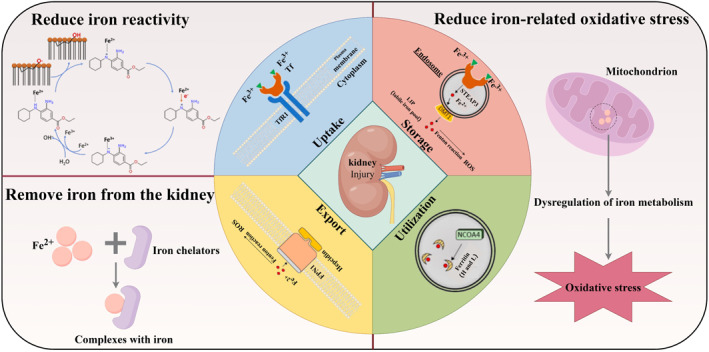
Schematic illustration of acute kidney injury and iron metabolism (By Figdraw). Image for iron metabolism, including uptake, storage, utilization, export: Reproduced with permission.^[135]^ Copyright 2022, International Journal of Molecular Sciences. Image for reducing iron reactivity: Reproduced with permission.^[111]^ Copyright 2020, Redox Biology.

This review delves into the intricate workings of renal iron metabolism and underscores its significance in both the treatment and prevention of AKI. It offers an in‐depth exploration of the dynamic relationship between AKI and iron metabolism, encompassing the dysregulation observed in AKI and the instances where iron metabolism disorders lead to AKI (Scheme 1). Furthermore, the review meticulously assesses a range of current therapeutic strategies that target iron metabolism for the management of AKI, with the goal of identifying and advancing more effective treatment approaches for patients suffering from this critical condition.

## 2 IRON METABOLISM

**FIGURE 1:**
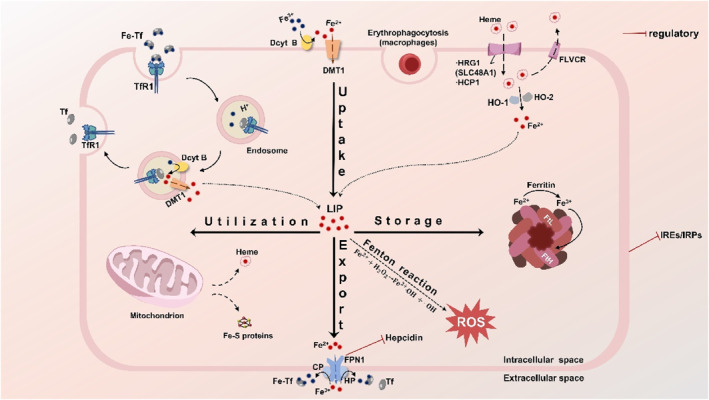
Systemic and cellular iron metabolism. The process of iron metabolism in cells is shown in Figure 1. Most cells acquire non‐heme iron through the process of endosome mediated by transferrin receptor 1 (TfR1), which binds to transferrin. In the endosomal environment, iron is initially separated from transferrin and is later reduced to Fe^2+^ by duodenal cytochrome B (Dcyt B). Post reduction, it is conveyed into the cytoplasm by the divalent metal transporter 1 (DMT1), while transferrin and its receptor TfR1 are recycled back into the cytoplasm. Transferrin and TfR1 are recycled back to the plasma membrane. DMT1, after reduction by Dcyt B and possibly other iron‐reductase enzymes, can also absorb iron from the diet at the apical membrane. Heme is shuttled by heme carrier protein 1 (HCP1) and/or heme‐responsive gene protein 1 (HRG1), and upon degradation by heme oxygenase 1 (HO‐1) and heme oxygenase 2 (HO‐2), it releases Fe^2+^. In an alternative mechanism, iron is exported together with heme by the feline leukemia virus subgroup C receptor (FLVCR), serving as a heme exporter. Other means of iron acquisition, such as macrophages obtaining heme iron from red blood cells, are also symbolically represented. The iron absorbed is allocated to the labile iron pool (LIP), which is a component of the body's iron uptake system. The level of the LIP is maintained by the equilibrium between the processes of iron uptake, utilization, storage, and export, thereby averting the risk of iron overload. The LIP can transport iron to the mitochondria, which are primarily responsible for the biosynthesis of heme and iron‐sulfur clusters. The segment of the LIP that is not allocated to metallization can be extruded from the cell via ferroportin‐1 (FPN1), where it is transformed into Fe^3+^ by the oxidation facilitated by ceruloplasmin (CP) and hephaestin (HP), or it can be accumulated within ferritin, comprised of the ferritin heavy chain (FtH) and the ferritin light chain (FtL). If there is too much iron in the LIP, the abnormal accumulation of free iron catalyzes the Fenton reaction, producing a large number of destructive hydroxyl radicals and other ROS, which can exacerbate cellular lipid peroxidation, and damage DNA, proteins, and cell membranes. In the regulation of systemic iron homeostasis, hepcidin plays a pivotal role, and FPN is regulated by hepcidin. Iron homeostasis within the cellular environment is primarily achieved by the influence of iron‐responsive elements (IREs) and the regulatory function of iron regulatory proteins (IRPs).

Iron ranks as one of the most plentiful elements on Earth, holding the position of the fourth most abundant constituent in the Earth's crust.^[20]^ Iron is a crucial micronutrient that is necessary for the regular physiological functioning of nearly all recognized living organisms.^[21]^ In the realm of biological processes, iron is a key element that contributes to the transportation of oxygen through hemoglobin, the oxygenation of muscles via myoglobin, the synthesis of proteins within the mitochondria's respiratory chain, and the formation of DNA by the enzyme ribonucleotide reductase.^[22]^ The body maintains a dynamic balance of systemic iron, which involves continuous absorption, utilization, storage, and recycling, known as iron homeostasis (Figure 1). This equilibrium is vital for healthy growth, development, and metabolic processes. Disruptions in iron homeostasis can result in the emergence of conditions associated with iron metabolism, encompassing both iron overload disorders and iron deficiency (ID) diseases.[^23^, ^24^, ^25^, ^26^]

Iron overload arises when an excess of iron accumulates in the body, posing a potential toxicity risk to the kidneys and other vital organs. Elevated levels of free iron are capable of catalyzing the Fenton reaction, producing a large amount of destructive hydroxyl radicals and other reactive oxygen species (ROS), further intensifying cellular lipid peroxidation reactions, damaging DNA, proteins, and cell membranes, and even leading to the occurrence of ferroptosis.^[27]^ Ferroptosis, a term first introduced by Dr. Brent R. Stockwell from Columbia University in 2012, is a novel form of iron‐dependent cell death that is not the same as apoptosis, necrosis, and autophagy.^[28]^ In the course of cellular ferroptosis, there occurs a buildup of substantial quantities of iron ions, which is accompanied by the oxidation of lipids and an escalation in the levels of ROS. Concurrently, there are alterations in the genes that govern the maintenance of iron balance and the metabolism of lipid peroxidation.^[29]^

When the body does not receive enough iron to fulfill its requirements or to make up for losses due to physiological or pathological factors, depletion of body iron stores leads to ID. ID has two main forms: one is absolute ID, where the body's iron stores are reduced; the other is functional ID, where the body's iron reserves are either normal or elevated, yet there is an insufficient delivery of iron to the bone marrow.^[30]^ Despite the absence of anemia, ID alone can result in feelings of weakness and can intensify chronic illnesses, which in turn can raise the incidence of other diseases and the risk of death. Prevalence rates of ID are notably high in several chronic disease groups. It is estimated that 37%–61% of chronic heart failure patients, 24%–85% of those with chronic kidney disease, and a broad spectrum of 13%–90% of individuals with inflammatory bowel disease are affected by this condition.^[31]^

### 2.1 Iron uptake

Under normal conditions, the body can only obtain iron from external sources through food, with the proximal duodenum being the primary absorption site for this iron. Iron in food mainly exists in the form of heme iron and non‐heme iron,^[32]^ and non‐heme iron can be further divided into simple inorganic iron, organic chelated iron, and iron from plant and animal proteins. Accordingly, animal organisms have also evolved absorption pathways that match both heme and non‐heme iron.^[33]^

Heme iron is absorbed at a higher efficiency rate than non‐heme iron, and it is absorbed in the small intestine. However, the exact mechanisms and proteins responsible for heme transport are not fully understood. Studies suggest that it is likely absorbed through specific transporters rather than by passive diffusion.^[34]^ Currently, two transporters are believed to be involved in the absorption of heme iron: one is HCP1,^[35]^ and another possible transport carrier is heme‐responsive gene protein HRG‐1, also known as SLC48A1. HCP1 is a transporter expressed on the luminal surface of intestinal cells, which not only transports heme but also other substrates such as folic acid.^[36]^ HRG1 is a heme transporter that can be used for the heme‐iron cycle in macrophages.^[37]^ Once heme enters the cell, heme oxygenase‐1 (HO‐1) and ‐2 (HO‐2) mediate the release of ferrous iron from heme into the cytosolic matrix.^[38]^

Non‐heme iron in the body is mainly absorbed from food through the duodenum. In addition, a portion of the body's iron comes from the release of iron by macrophages in the spleen that engulf aging red blood cells.^[39]^ Under physiological conditions, the duodenum absorbs iron from the diet based on the body's overall iron requirements, facilitated by Dcyt B^[40]^ and reducers such as ascorbate.^[41]^ Fe^2+^ is transported into the cell by DMT1 (also referred to as SLC11A2) through a proton‐coupled process across the apical membrane of small intestinal epithelial cells, a step that follows the reduction of Fe^3+^ in the duodenal lumen to Fe^2+^.^[42]^ Once inside the epithelial cells, Fe^2+^ is oxidized to Fe^3+^ by ceruloplasmin (CP) and hephaestin (HP).^[43]^ It is then released into the bloodstream from the basal membrane by Ferroportin 1 (FPN1) to participate in the body's circulation.^[44]^

### 2.2 Iron utilization

Absorbed iron first enters the liver and is then transported to tissues, with most of it being used for red blood cell production. The remaining iron enters various organs through the bloodstream. The primary form of iron transport into cells within the blood is through transferrin (Tf).^[45]^ After Fe^2+^ is oxidized to Fe^3+^ by ceruloplasmin, it binds to transferrin Tf to form an iron‐transferrin complex (Fe‐Tf), which can then bind to the TfR1.^[45]^ The complex enters the cell through the endosome as a small vesicle. Subsequently, the pH of the vesicle changes, reducing Fe^3+^ back to Fe^2+^,^[46]^ which is then transported into the cytoplasm by DMT1 for utilization. Generally, iron that enters the cell is directed to the mitochondria, where it is primarily responsible for the biosynthesis of heme and iron‐sulfur clusters.^[47]^ Excess iron is stored in the form of Fe^3+^ either in ferritin or transferred outside the cell by FPN1.^[48]^

### 2.3 Iron storage

Catalytic iron, also referred to as labile iron, constitutes a dynamic reservoir of iron that is present in both extracellular and intracellular spaces, termed the LIP.^[49]^ It does not bind to transferrin but loosely associates with albumin or low molecular weight metal‐binding groups such as citrate, acetate, malate, phosphate, and adenosine nucleotides.[^50^, ^51^] A portion of the intracellular ferrous iron is incorporated into the LIP, while the rest is stored in ferritin. Ferritin is the primary form of iron storage within cells and an essential component of cellular iron homeostasis.^[52]^ It encapsulates iron atoms within a spherical shell composed of 24 subunits arranged around a large central cavity, isolating potentially damaging iron ions from intracellular structures while forming an intracellular iron reservoir for biological use. A single ferritin molecule can accommodate up to 4500 iron atoms.^[53]^ Ferritin is composed of peptide subunits, which consist of the ferritin heavy chain (FtH) and the FtL.^[54]^ FtH possesses iron oxidase activity, oxidizing Fe^2+^ to Fe^3+^, which allows for better binding to ferritin.^[55]^ FtL lacks enzymatic activity and cannot oxidize iron ions or take up iron ions. However, FtL serves as the nucleation site for iron ions because it has more carboxyl groups on the surface of the ferritin cavity. Experiments have shown that FtL's ability to induce iron ion nucleation is stronger than that of FtH, and the heavy and light chains have a synergistic effect on iron ion uptake.^[56]^

### 2.4 Iron export

The receptor for Feline Leukemia Virus Subgroup C, known as FLVCR, functions as a protein responsible for the export of heme.^[57]^ In macrophages, the FLVCR aids in the recycling of heme‐iron, indicating that systemic iron balance is regulated by the transport of heme‐iron through FLVCR as well as by the more familiar iron trafficking pathways.^[58]^

FPN1 is recognized as the sole iron‐exporting protein in mammals, located on the cell membrane, and it only allows iron to be transported out of the cell in the form of Fe^2+^.^[44]^ It binds to intracellular Fe^2+^ and, with the action of ceruloplasmin, converts it to Fe^3+^, promoting its binding with transferrin and transportation throughout the body.^[59]^

### 2.5 Regulation of iron homeostasis

**FIGURE 2:**
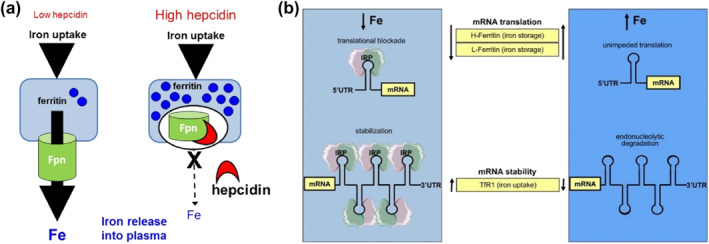
(a) Hepcidin functions via effects on FPN mediated iron efflux. (b) Coordinate iron‐dependent regulation of ferritin and TfR1 mRNA expression by IRE/IRP interactions.^[60]^ Reproduced with permission. Copyright 2014, Wilkinson and Pantopoulos. FPN, ferroportin; IRE, iron‐responsive element; IRP, iron regulatory protein; mRNA, messenger ribonucleic acid; TfR1, transferrin receptor 1.

Homeostasis of iron metabolism in the body is the result of fine regulation at multiple levels and from various perspectives by cells and systems.^[60]^ Hepcidin is crucial in the control of systemic iron balance within the body. It is a small antimicrobial peptide hormone primarily produced and secreted by the liver, with minor expression also found in macrophages, adipocytes, and other cells.^[61]^ The only well‐documented physiological activity of hepcidin is its regulation of the iron transport protein, FPN. Hepcidin binds to FPN (Figure 2a), leading to its endocytosis and proteolysis, mainly in lysosomes. This interaction leads to a diminished release of iron from macrophages and intestinal epithelial cells, which in turn causes a decline in the levels of iron found in the serum.^[44]^ Since FPN mediates iron export in all cells, the regulation by the hepcidin/FPN axis affects intestinal epithelial cells that absorb iron from the diet, macrophages that recycle iron from aging red blood cells or other cells, and hepatocytes that are involved in iron storage.[^62^, ^63^]

Iron homeostasis within cells is chiefly regulated by two key components: iron response elements (IREs) and IRPs (Figure 2b). These elements and proteins perform their regulatory functions after the transcription phase of gene expression.^[64]^ IREs are highly conserved nucleotide sequences found within the target mRNA, messenger ribonucleic acid (mRNA), and IRPs are cytosolic soluble proteins composed of RNA loops with the consensus sequence CAGUG, divided into two subtypes, IRP1 and IRP2.^[65]^ IRPs can bind to the RNA stem‐loops containing IREs in the untranslated regions to manipulate the translation of target mRNAs.^[60]^ However, iron can bind to IRPs, causing them to separate from IREs and alter the translation of target transcripts. In iron‐deficient states, the excessive binding of iron to IRPs leads to conformational changes, allowing IRPs to bind to the IREs of TfR1 mRNAs, inhibiting their degradation, increasing iron uptake, and suppressing the translation of ferritin, thereby blocking iron storage and release.^[66]^ Conversely, when iron is abundant within the cell, IRPs do not bind to IREs, which increases the utilization, storage, and release of iron within the cell and reduces iron uptake, maintaining iron levels within an appropriate concentration range.^[67]^

However, a critical and immediate challenge lies in the need to further elucidate the complex dialog that occurs between systemic and cellular iron control systems. It is essential to continually acknowledge and address the reality that there are fundamental questions in the cell biology of iron that have yet to be satisfactorily answered.

## 3 THE PATHOPHYSIOLOGY OF AKI RELATED TO IRON HOMEOSTASIS

The kidney is among the most essential organs for waste elimination in the human body, with the nephron being its fundamental structural and functional component.^[68]^ When blood flows through the glomerulus, all small molecular substances in the blood, except for blood cells and large molecular plasma proteins, can pass through the glomerular filtration membrane into the Bowman's capsule. As the renal tubular fluid flows through various segments of the renal tubules, substances in the tubular fluid can be selectively reabsorbed partially or entirely by the renal tubules, while metabolic products are excreted through the final urine. The biological basic form of iron in plasma is Fe‐Tf,^[69]^ with a molecular weight of 78 ku, which is widely believed to exceed the maximum value of glomerular filtration.^[70]^ Therefore, it is commonly thought that the glomerulus cannot filter iron, and as a result, research related to renal iron metabolism is often overlooked. However, in recent years, research has revealed a multitude of transport proteins and regulatory mechanisms that participate in renal iron metabolism.^[71]^

Glomerular endothelial cells, mesangial cells, and proximal and distal tubular epithelial cells all express TfR1. Fe^3+^ bound to transferrin in the circulation is filtered through the glomerulus via TfR1 and enters the renal tubular lumen.^[72]^ Under physiological conditions, the glomerulus filters 10–30 μg of transferrin‐bound iron per day.^[73]^ This value may be underestimated as the possibility of transferrin being reabsorbed in the distal convoluted tubule and collecting duct through the neutrophil gelatinase associated lipocalin receptor (NGALR) was out of consideration. After passing through the glomerular filtration, iron is nearly entirely reclaimed in both the proximal and distal tubules. Here, it is sequestered within ferritin and employed by the cells of the renal tubular epithelium. Additionally, the proximal tubule can absorb transferrin‐bound iron through endocytosis mediated by megalin and cubilin.^[74]^ Most knowledge about renal iron handling is related to the renal tubular epithelial system. Exclusively on the apical membrane of these polarized cells is where iron uptake is detected. Furthermore, an increased presence of Tf in urine is a common finding in patients with renal tubular dysfunction.^[73]^ Furthermore, renal tubular cells express multiple proteins related to iron metabolism, including DMT1, FPN1, and hepcidin. Wareing et al.^[75]^ have demonstrated that DMT1 may play a major role in renal iron reabsorption, with a significant portion of serum iron being ultrafiltered by the glomerulus, and most of the iron filtered by the glomerulus being reabsorbed. Wolff et al.^[76]^ believe that anemia increases the expression of FPN1 in renal segments, leading to its redistribution to the apical membrane, where FPN1 exports iron from renal proximal tubular cells, reducing intracellular iron concentration and facilitating iron excretion in anemic animals. Kulaksiz et al.^[77]^ showed that hepcidin is produced in the renal epithelial tubular and ductal cells and may be released into the urine lumen, indicating a regulatory role for hepcidin in the kidney or urinary tract. Renal tubular epithelial cells in both the proximal and distal segments possess HO‐1 for the metabolism of heme catabolism and ferritin complexes to store iron^[78]^; however, it is exclusively the proximal renal tubular epithelial cells that exhibit the expression of FPN on their basolateral membrane to facilitate iron export, a process that can be suppressed by hepcidin present in the bloodstream.^[79]^ Despite its importance, our understanding of the renal handling of iron remains limited. Iron is subject to glomerular filtration and subsequent reabsorption in the kidney, a process that holds considerable physiological significance and could offer therapeutic opportunities. It is imperative that future research places a greater emphasis on exploring the intricate dynamics of iron metabolism across organ systems and the complex intercellular interactions that govern these processes.

**FIGURE 3:**
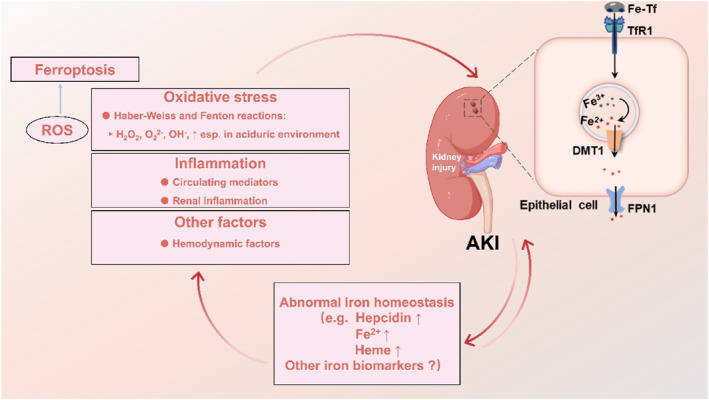
The link between AKI and the abnormal iron homeostasis. It depicts that the renal tubular epithelial cells express certain genes related to iron metabolism, such as DMT1, FPN1, and TfR1. It also outlines that when AKI occurs due to the production of excessive reactive oxygen species (ROS) causing oxidative stress, inflammation, and other factors, there is a dysregulation in the expression of renal iron metabolism genes. Conversely, abnormal iron homeostasis can exacerbate the occurrence of AKI either directly or indirectly through oxidative stress, inflammation, and other factors. AKI, acute kidney injury; DMT1, divalent metal transporter 1; FPN1, ferroportin 1; ROS, reactive oxygen species; TfR1, transferrin receptor 1.

The connection between the kidney and iron metabolism is closely intertwined (Figure 3), not only because the kidney serves a critical function in maintaining iron homeostasis but also because renal injury may be linked to the disruption of iron homeostasis.^[73]^ When AKI occurs, due to ischemic or toxic damage to the kidney, there is an increased release of intracellular unstable iron, which disrupts iron homeostasis and leads to oxidative damage.^[80]^ Conversely, iron overload, caused by various factors, can also cause damage to the kidneys. During iron overload, a large amount of iron enters the renal tubules, exposing the kidneys to toxic levels of free and bound iron (such as transferrin). Excessive iron enhances oxidative stress reactions through the Fenton reaction, leading to renal injury.^[81]^

Throughout the last 30 years, imbalances in iron homeostasis and the cytotoxic effects of iron have been acknowledged as contributors as well as outcomes of renal damage.

### 3.1 Disorders of iron in AKI

The kidney plays a significant role in regulating iron homeostasis.^[71]^ On one hand, this may be to prevent renal damage from iron toxicity, and on the other hand, it may be to prevent iron loss through the urinary tract, maintaining iron levels within the normal physiological range to meet the body's demand for iron. This ensures that adequate iron is available for the production of red blood cells and for the fulfillment of other important physiological functions.

Under physiological conditions, iron ions within renal tubular epithelial cells are bound to transferrin and ferritin and exist stably in the form of Fe^3+^.^[82]^ However, when the kidney is in a pathological state such as ischemia/reperfusion injury or inflammatory responses, on one hand, Fe^3+^ readily undergoes electron transfer to form Fe^2+^, and on the other hand, the intracellular iron chelator FtH is extensively degraded, increasing the Fe^2+^ content in cells.^[83]^ Through the Fenton reaction, Fe^2+^ can lead to the generation of significant levels of ROS, including superoxide anions and hydrogen peroxide. These ROS have the potential to cause damage to cellular components like lipids, nucleic acids, and proteins, both structurally and functionally, and may even result in ferroptosis.^[84]^ Understanding the changes in iron metabolism‐related indicators during the development of AKI is beneficial for the development of novel diagnostic and prognostic markers for the early identification of AKI.^[85]^

The search for early AKI biomarkers has primarily focused on proteins related to the progression or development of kidney injury, such as NGAL, KIM‐1, and IL‐8.[^86^, ^87^, ^88^] But the existing diagnostic approaches are characterized by their invasive nature and their limitation in providing tissue‐specific details to delineate the extent and condition of pathological changes. To overcome these shortcomings, Zeng et al.^[89]^ developed an artemisinin‐derived probe, designated as Art‐Gd. This probe leverages the redox reactivity of Fe^2+^ as a distinct chemical target for the detection of ferroptosis through contrast‐enhanced magnetic resonance imaging (feMRI). This innovative technique is able to reveal MRI contrast indicative of AKI up to 24–48 h earlier than the conventional clinical tests used for AKI assessment. Utilizing the Art‐Gd probe, feMRI can identify the abnormal intracellular concentrations of Fe^2+^ within the cardiac and renal cells of mice subjected to AKI as a result of anticancer drug administration.

However, another unique protein has been identified, FtL 25 (ferritin‐25), which may be associated with the prevention of AKI.^[90]^ In another prospective nested case‐control study (*N* = 250),^[91]^ the continuous urinary proteome of 22 AKI patients and 22 non‐AKI patients before, during, and after cardiopulmonary bypass (CPB) surgery were compared, and three new biomarkers for kidney function after cardiac surgery were identified: NGAL, hepcidin 25, and alpha‐1 microglobulin (α1MG). Naohisa and others^[92]^ had developed a semi‐quantitative system for measuring serum hepcidin‐25 and have utilized it in clinical cases involving iron overload, ID, and both acute and chronic inflammation. The introduction of this novel semi‐quantitative assay for serum hepcidin is expected to broaden the scope of research into iron homeostasis under various human physiological and pathological conditions. Scindia et al.^[93]^ demonstrated that the levels of non‐heme iron in the liver and spleen were significantly lower after ischemia‐reperfusion injury (IRI), and renal IRI led to a significant upregulation of hepatic hepcidin gene expression and increased serum hepcidin levels. However, further research is needed to clarify the source and dynamics of ferritin‐25 in renal IRI.

Cisplatin is a commonly utilized medication in chemotherapy for solid cancers; nevertheless, it is associated with kidney toxicity as a side effect. Among patients receiving cisplatin therapy, there is a 30% chance of developing AKI. Kim et al.^[94]^ illustrated that cisplatin can increase renal iron levels in rats and mice and promote iron‐catalyzed oxidative damage, thereby accelerating renal ferroptosis.

Investigating the potential correlation between urinary catalytic iron and AKI occurrence after cardiac surgery, Akrawinthawong et al.^[95]^ observed that urinary catalytic iron levels were notably increased 8 h after the procedure in patients with AKI. This is in stark contrast to creatinine levels, which only began to show significant differences 12 h postoperatively. Therefore, urinary catalytic iron may predict AKI earlier than creatinine after cardiac surgery. Choi et al.^[96]^ found that a high transferrin saturation level 1 h after extracorporeal circulation is an independent predictor of AKI. Animal‐based research has revealed that injecting non‐bound transferrin intraperitoneally can diminish the levels of free iron in the circulation of mice that have experienced ischemia‐reperfusion, halt the formation of superoxides in the kidneys, and decrease the inflammation caused by neutrophil infiltration,^[95]^ indicating that transferrin saturation level can serve as an early predictor of AKI after cardiac surgery, and non‐coupled transferrin can be used to enhance endogenous iron‐binding capacity. Zhao et al.^[85]^ discovered that the ratio of urinary hepcidin to urinary creatinine is a better predictor of AKI incidence than serum creatinine.

These findings suggest that some iron metabolism‐related genes may be promising biomarkers for AKI. In a groundbreaking study conducted by Leaf et al.,^[97]^ a thorough evaluation of circulating iron parameters was performed on critically ill patients with AKI who were in need of RRT. The researchers delved into various iron‐related indicators to ascertain their correlation with the clinical outcomes of these patients. Their findings reveal a significant association between multiple iron parameters and the risk of mortality in this patient population. Notably, the study highlights the plasma catalytic iron concentration, an indicator of the reactive iron species present in the blood, and the levels of hepcidin, a key regulatory hormone in iron metabolism. Both of these parameters were found to be predictive of adverse outcomes in critically ill AKI patients undergoing RRT.

### 3.2 The role of disordered iron metabolism in AKI

It has long been posited that iron may act as a probable facilitator of oxidative stress and cellular harm. Unbound iron possesses catalytic capabilities, enabling it to facilitate the generation of deleterious free radicals via the Haber‐Weiss and Fenton reactions, which in turn results in oxidative cellular injury.^[98]^

In animal models of AKI, catalytic iron is associated with renal toxicity caused by various types of injury, including ischemia/reperfusion,^[99]^ aminoglycosides,^[100]^ and rhabdomyolysis.^[101]^

Ferritin has a strong iron‐clearing capability. In the proximal tubules, the specific absence of FTH exacerbates AKI induced by rhabdomyolysis or cisplatin, reducing the survival rate in mice.^[83]^ In contrast, inducing the expression of FTH in the kidney can mitigate renal injury by enhancing iron chelation within ferritin, reducing oxidative stress markers, and alleviating the decline in renal function caused by ischemia‐reperfusion, demonstrating a protective effect in AKI mouse models.[^83^, ^102^]

Compared with wild‐type mice, hepcidin‐deficient mice have an increased susceptibility to IRI. Reconstitution of hepcidin in hepcidin‐deficient mice with exogenous hepcidin can induce hepatic iron chelation, reduce the decrease of renal H‐ferritin, and decrease renal oxidative stress, apoptosis, inflammation, and tubular damage.^[93]^ Within AKI animal models, the administration of hepcidin from an external source can reestablish iron equilibrium by elevating the expression of ferritin and the process of iron chelation, thereby protecting the kidney and reducing renal oxidative stress and inflammation.[^93^, ^103^] The findings from studies conducted by Leaf et al.^[104]^ and Lele et al.^[105]^ have been successfully extended from animal models to human subjects, yielding consistent results with previous research. David's team conducted a single‐center, prospective, non‐consecutive cohort study on 121 critically ill patients admitted to the intensive care unit (ICU) between 2008 and 2012. The study found that higher plasma catalytic iron levels upon arrival at the ICU were significantly associated with increased risk of death/re‐treatment, in‐hospital and 30‐day mortality rates, incidence and severity of AKI, and prolonged hospital stays. Furthermore, Suhas and colleagues measured the serum catalytic iron levels of 806 patients with acute coronary syndrome (ACS) undergoing cardiac surgery with contrast agent exposure. Since iodinated radiocontrast agent exposure is a significant cause of AKI in patients undergoing percutaneous coronary intervention in the context of ACS, their results suggest that high baseline catalytic iron levels are associated with higher mortality rates. Additionally, further increases in catalytic iron levels may be one of the potential pathophysiological mechanisms explaining the high mortality associated with contrast‐induced nephropathy.

Ongoing clinical trials are assessing the potential applications of hepcidin agonists across a range of clinical conditions.^[106]^ Conversely, the delivery of hepcidin is viewed as a prospective treatment approach for preventing and managing AKI within intensive care units.^[107]^

In addition, an increasing amount of data suggests that ferroptosis is involved in AKI.^[108]^ Under normal physiological conditions, redox reactions in lipids and oxylipids serve as highly diverse signals for various processes. However, if redox metabolism overwhelms the body's antioxidant capacity, it can trigger ferroptosis. The kidneys, due to the high catalytic activity of iron‐containing proteins in their oxygen consumption rate, are particularly susceptible to redox imbalances, making both redox dysregulation and ferroptosis more likely to occur in AKI. Glutathione peroxidase Gpx4 is a central regulator of ferroptosis, which inhibits ferroptosis in a glutathione‐dependent manner.^[109]^ Friedmann Angeli JP and colleagues have demonstrated that the inactivation of Gpx4 induces acute kidney failure in mice.^[110]^ Ferrostatin‐1 (Fer‐1) is a ferroptosis inhibitor that preserves kidney function and reduces histological damage, oxidative stress, and renal tubular cell death in a folic acid (FA)‐induced AKI mouse model.^[18]^ Targeting the molecules involved in ferroptosis could provide new therapeutic strategies for AKI, although further in‐depth research is needed, of course.

## 4 APPROACH OF TARGETED IRON METABOLISM THERAPY FOR AKI

It is significant to recognize that the intricacy of iron homeostasis offers numerous avenues for therapeutic intervention. Understanding the relationship between the kidney and iron metabolism, it is currently believed that kidney injury caused by iron can be prevented or treated by reducing iron reactivity, removing iron from the kidney, or reducing iron‐related oxidative stress (Table 1). While targeted strategies in iron metabolism hold promise for the treatment and understanding of AKI, the specific benefits and limitations of these approaches are not yet clear‐cut. There is an anticipation for more extensive research to be conducted on the interplay between iron metabolism and AKI with the aim of expanding our current knowledge and improving the technological strategies available for addressing renal damage.

**TABLE 1:** Avenues for therapeutic intervention in AKI.

Classification	Mechanism	Name	References
Reduce iron reactivity	By reducing the reactivity of iron through the action of drugs, the process of ferroptosis can be inhibited, thereby achieving the effect of mitigating AKI	Fer‐1	[111]
		Lip‐1	[112]
		Polydatin	[113]
		Quercetin	[114]
Remove iron from the kidney	Complexing with circulating iron and, to a lesser extent, cellular iron, the chelating agents facilitate iron excretion through urine and feces, aiding in the management of iron overload. The vast majority of evidence indicates that iron chelators can substantially reduce the impact of AKI in animal models	DFO	[115]
		DFP	[116]
		DFX	[117]
		QDC	[118]
Reduce iron‐related oxidative stress	Antioxidants are believed to modulate ROS, iron metabolism, and lipid peroxidation. The use of antioxidant therapy may reduce iron‐induced tissue damage caused by oxidative stress	Vitamin E	[119]
		Entacapone cytochrome P450	[120]
		Mackinawite nano enzyme	[121]
		Fe‐Cur CPNs	[122]
			[123]

Abbreviations: AKI, acute kidney injury; CPN, coordination polymer nanodots; DFO, deferoxamine; DFP, deferiprone; DFX, deferasirox; QDC, quantum dot drug conjugates.

### 4.1 Reduce iron reactivity

It is recognized that Fe^2+^ acts as a catalyst in the Fenton reaction, thereby generating substantial quantities of ROS, and when the ROS level is excessively high, it will directly or indirectly destroy the damage cellular structures and functions, leading to a form of cell death known as ferroptosis.

Hence, the strategy involves employing drugs to diminish iron reactivity, thereby inhibiting ferroptosis and consequently easing the condition of AKI. Fer‐1 can scavenge free radicals generated from lipid peroxidation and other rearrangement products formed by Fe^2+^, thus inhibiting ferroptosis.^[111]^ Although Fer‐1 prevents folate‐induced AKI, its instability in vivo limits its application.^[18]^ Similar to Fer‐1, liproxstatin‐1 (Lip‐1) is a specific ferroptosis inhibitor identified through high‐throughput screening that does not interfere with other types of cell death pathways but has greater stability in vivo than Fer‐1.^[112]^ However, the specific mechanisms behind LIP‐1 are not yet clear, and whether the results from in vitro studies can be extended to in vivo systems remains an open question.

In addition, traditional Chinese medicine (TCM) has unique advantages in the prevention and treatment of AKI. In recent years, more and more research have focused on the effects and mechanisms of active components of TCM in alleviating AKI by regulating ferroptosis. Polydatin,^[113]^ extracted from the Chinese medicine Polygonum cuspidatum, is a polyphenolic active component that shows potential therapeutic effects in various kidney diseases with antioxidant stress, anti‐inflammatory, and anti‐fibrotic properties. Studies have found that polydatin alleviates cell death induced by the ferroptosis inducer erastin in human renal tubular epithelial cells (HK‐2) in a dose‐dependent manner, significantly inhibiting ferroptosis by reducing iron overload and increasing GPX4 activity. Quercetin, a natural flavonoid, acts as a ferroptosis inhibitor to suppress ferroptosis in renal proximal tubular epithelial cells and simultaneously reduces the chemotaxis of macrophages induced by ferroptosis in AKI.^[114]^ Although TCM holds great potential as a therapeutic target, most research to date has focused on the active components of TCM. Given the complexity of TCM ingredients, this approach may not fully harness the effects of TCM, and the long‐term efficacy of these active components has yet to be evaluated.

### 4.2 Remove iron from the kidney

Iron chelators can form complexes with iron in the circulation and within cells to varying degrees, thereby promoting the excretion of iron through urine and feces. The majority of evidence supports that iron chelators can greatly lessen the severity of AKI in animal models.[^124^, ^125^] Current iron chelators include Deferoxamine (DFO),^[115]^ Deferiprone (DFP),^[116]^ and Deferasirox (DFX),^[117]^ but their effectiveness in iron removal is limited when used alone, restricting their broad application. Feng et al.^[126]^ designed a novel class of DFO with an adjustable skeleton and flexibility, which effectively removed excess iron in vitro and in a mouse model of iron overload. It demonstrates a stronger ability to bind iron than traditional chelating agents through two molecular oxygens in the phenolic hydroxyl groups and one nitrogen atom in the amine in a chemical ratio of 2:1. However, the study only showed significant efficacy in improving iron‐related damage and did not further develop treatments. DFP, a newer oral iron chelator, has also been proven to mitigate AKI in two studies: a cisplatin induced rat AKI model^[127]^ and an aluminum chloride‐induced mouse AKI model.^[128]^ In the latter study, the combination of DFP and DFO treatment provided stronger renal protection than DFP alone. Other iron‐binding compounds, including haptoglobin and neutrophil gelatinase‐associated lipocalin, have been shown to alleviate the severity of renal injury after IRI.[^129^, ^130^] Menasché et al.^[131]^ assessed the impact of intravenous DFO as opposed to a placebo in 24 adults undergoing cardiac surgery with CPB. While the study's size was inadequate to identify any variances in AKI occurrence between the two groups, it was observed that neutrophils from patients who received DFO generated a lower quantity of superoxide radicals in comparison to those in the control group. More recently, nanotechnology‐based iron chelators have been engineered for the specific extraction of iron in the renal context. Carbon quantum dots (CDs) show great promise in biomedical applications due to their high stability, abundant and low‐cost sources, ease of large‐scale production, and unique physicochemical properties. Quantum dot drug conjugates (QDC) with high ROS scavenging activity and favorable renal‐specific biodistribution were designed and prepared by Zhu et al.^[118]^ These QDCs were composed of CDs, DFO with iron‐chelating capability, and polyethylene glycol for prolonged body retention. They were developed to alleviate AKI induced by chemotherapy drugs. However, similarly, this study only provides a new paradigm where the developed drug can achieve dual targeting of both removing pathologically unstable iron species and eliminating excess ROS produced in the kidney, addressing both symptoms and the root cause. However, it does not specify the clinical application of the new drug or its assessment for subsequent treatments.

### 4.3 Reduces iron‐related oxidative stress

Antioxidants are believed to regulate ROS, iron metabolism, and lipid peroxidation, and their use in treatment may reduce iron‐induced tissue damage caused by oxidative stress.^[132]^ Vitamin E,^[119]^ a fat‐soluble antioxidant naturally found in foods such as plant seeds, vegetables, and eggs, includes α‐tocopherol as its main component, which can reduce cellular lipid peroxides. Entacapone, acting as a specific inhibitor of catechol‐O‐methyltransferase (COMT), has been employed for a long time as an additional treatment option for individuals with Parkinson's disease.^[133]^ It has been discovered in recent research^[120]^ that Entacapone could play a role in alleviating AKI by curbing lipid peroxidation and iron accumulation, which is thought to be due to its capacity to strengthen antioxidant systems. Mishima et al.^[121]^ screened a series of cytochrome P450 substrate compounds and identified a group of drugs and hormones with lipid peroxyl radical scavenging activity, which inhibit lipid peroxidation by scavenging lipid peroxyl radicals that promote ferroptosis in various cell models and AKI mice. The use of lipid peroxyl radical‐specific free radical scavengers for treatment represents a promising pharmacological approach to inhibit lipid peroxidation, but it has only demonstrated therapeutic efficacy in acute disease models. Even if the drug has certain therapeutic effects, if the compound possesses chronic cytotoxic effects, long‐term use may also show harmful actions. In contrast to the natural antioxidants and antioxidant drugs discovered, the latest advances in nanotechnology have developed a class of nanomaterials with ROS scavenging capabilities that can be used for AKI antioxidant therapy. A novel mackinawite nanoenzyme (represented as GFeSNs) was developed by Xu et al.,^[122]^ which was synthesized from GSH and iron ions using a hydrothermal method. It shows broad‐spectrum ROS elimination activity against oxidative stress and excellent therapeutic efficiency in ROS‐induced AKI in vivo. Both in vitro and in vivo experiments have demonstrated that GFeSNs exhibit outstanding cellular protective effects against ROS‐induced damage at extremely low doses, and significantly improve the therapeutic outcomes of AKI. Zhang et al.^[123]^ added iron ions to a methanol solution of curcumin (Cur) and synthesized ultra‐small coordination polymer nanodots (CPNs) through a simple and robust method. After intravenous injection into AKI mice, the Fe‐Cur CPNs with effective renal retention can clear ROS in the kidneys after AKI and protect normal kidney function.

## 5 CONCLUSION AND PERSPECTIVE

This review article encapsulates the intricate mechanisms of iron metabolism in the kidney, encompassing the uptake, utilization, storage, and export of iron that constitute iron homeostasis, and highlights the potential significance of iron metabolism pathways in the treatment and prevention of AKI. It also delves into the interplay between AKI and iron metabolism, including the disruption of iron metabolism in AKI and the AKI triggered by abnormal iron metabolism. Besides these points, the article also consolidates findings from various research studies, collectively providing substantial evidence that iron plays a significant pathogenic role in AKI across both animals and humans, emphasizing that iron‐mediated toxicity is a prevalent mechanism underlying AKI. Furthermore, it provides a comprehensive review of current treatment strategies for AKI targeting iron metabolism, summarizing three main therapeutic approaches, with the aim of offering more rational treatment plans for future AKI patients.

Despite this, the understanding of the interplay between iron metabolism and AKI is considered a relatively new discovery that requires further validation through research. Moreover, several issues should be addressed in subsequent studies.

Firstly, current research on the interaction between iron metabolism and AKI comes from both in vivo and in vitro studies, but more research is needed to determine whether genes related to iron metabolism can serve as useful novel renal biomarkers in humans. We anticipate the emergence of additional biomarkers that can facilitate the prompt recognition of AKI in patients, enabling more efficient interventions and enhancement of patient conditions.

Refined experimental animal models are highly effective for in‐depth disease research, yet most can only partially simulate the progression of human diseases, a limitation evident in AKI models.^[134]^ Current AKI experimental models encompass zebrafish, rodents, and large animal models. Regrettably, previous studies have indicated that rodent models fail to accurately represent the disease progression of human AKI, constraining the translation of basic research based on these models into clinical practice. Therefore, more reliable animal models need to be explored, and clinical studies are also encouraged.

In addition, there is an urgent need for well‐designed, randomized, double‐blind, placebo‐controlled trials with adequate power to assess whether interventions targeting iron metabolism can reliably improve human AKI by targeting the iron regulatory pathways, providing clues for specific, localized, and effective treatment methods for the prevention and treatment of iron‐induced kidney damage. Despite the existence of the aforementioned challenges, which should be addressed in future research, we believe that focusing on the homeostasis of iron metabolism is essential and promising. As researchers conduct large‐scale studies on iron metabolism, clinicians should anticipate some attention and increasing hope.

## CONFLICT OF INTEREST STATEMENT

The authors declare no conflicts of interest.
